# Supplementary figures and images for: Association between serum periostin levels and the severity of arsenic-induced skin lesions
Source: PLoS One. 2023 Jan 4;18(1):e0279893. doi: 10.1371/journal.pone.0279893 (PMC9812306; doi:10.1371/journal.pone.0279893)

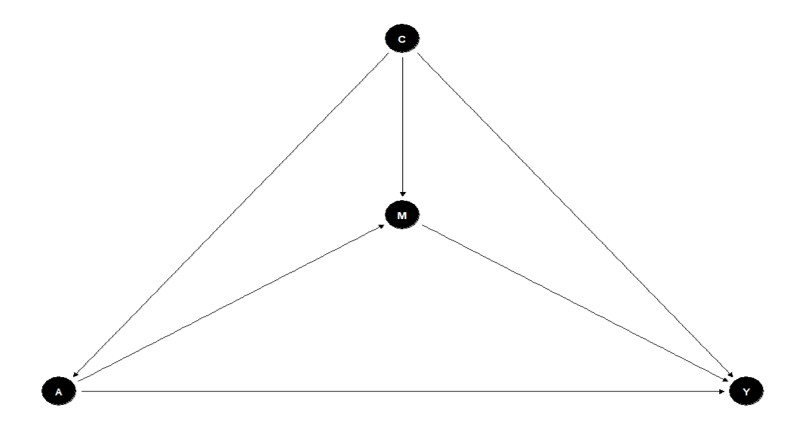

Supplement: S1 Fig — A (exposure): arsenic exposure metrics; M (mediator): periostin; C (confounders not affected by the exposure): age, sex, BMI, smoking, education; and Y (outcome): skin lesions. (TIF) [file pone.0279893.s001.tif]

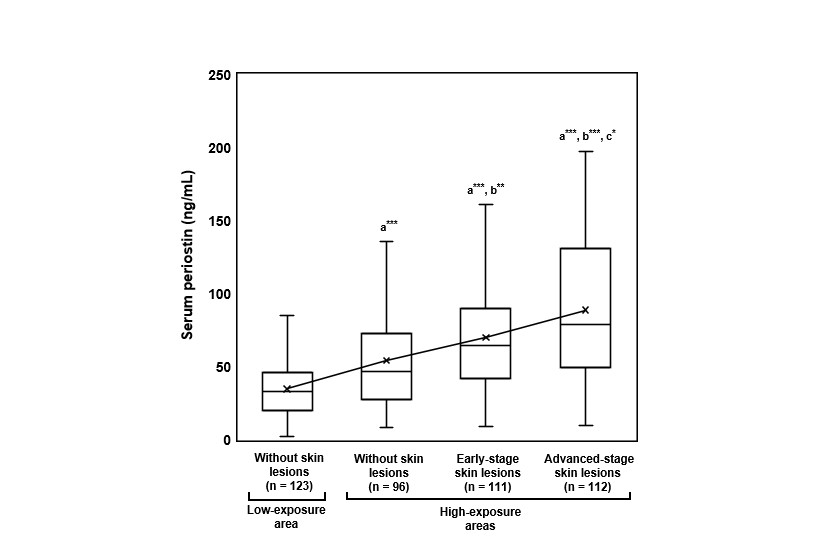

Supplement: S2 Fig — Comparison among the without skin lesions group in the low-exposure area and without, early- and advanced-stage skin lesions groups in the high-exposure areas. × indicates the mean and the straight line connecting the × indicates the mean line. Mean differences were assessed by using the Kruskal-Wallis test followed by Dunn-Bonferroni post hoc test between each skin lesions group. a, b, c Significant difference from without skin lesions group in the low-exposure area, without skin lesions group in the high-exposure areas, and early-stage skin lesions group, respectively. ***p < 0.001; **p < 0.01; *p < 0.05. (TIF) [file pone.0279893.s002.tif]
