# Supplementary material for: Association between serum periostin levels and the severity of arsenic-induced skin lesions
Source: PLoS One. 2023 Jan 4;18(1):e0279893. doi: 10.1371/journal.pone.0279893 (PMC9812306; doi:10.1371/journal.pone.0279893)
Supplement: S1 Table — (DOCX) [file pone.0279893.s003.docx]

**Table S1. Comparisons of arsenic exposure levels between the groups of participants with and without skin lesions.**

| **Parameters** | **All (n = 442)** | **Skin lesions** |  | ***p*-value** |
| --- | --- | --- | --- | --- |
|  |  | **No (n = 219)** | **Yes (n = 223)** |  |
| **Water As (µg/L)** | 65.92 (2.94, 205) | 3.20 (0.96, 82.73) | 167.94 (54.40, 263) | <0.001 |
| **Hair As (µg/g)** | 1.82 (0.47, 4.34) | 0.56 (0.24, 2.03) | 3.10 (1.59, 6.32) | <0.001 |
| **Nail As (µg/g)** | 3.83 (1.50, 10.12) | 1.83 (0.82, 3.78) | 7.26 (3.82, 14.10) | <0.001 |

Results are presented as median (25th percentile, and 75th percentile). Abbreviation: As, Arsenic. *p*-values were from the Mann-Whitney U test.
