# Supplementary material for: Association between serum periostin levels and the severity of arsenic-induced skin lesions
Source: PLoS One. 2023 Jan 4;18(1):e0279893. doi: 10.1371/journal.pone.0279893 (PMC9812306; doi:10.1371/journal.pone.0279893)
Supplement: S2 Table — (DOCX) [file pone.0279893.s004.docx]

**Table S2. Comparisons of the levels of type 2 cytokines and IgE in the different stages of skin lesions.**

| **Parameters** | **Without skin lesions** | **Early-stage skin lesions** | **Advanced-stage skin lesions** |
| --- | --- | --- | --- |
| **IL-4 (pg/mL)** | 31.02 (20.85, 42.35) | 41 (28.58, 50.40) a^***^ | 36 (28.10, 52.75) a^**^ |
| **IL-5 (pg/mL)** | 39.20 (34.75, 45.80) | 46 (38.95, 53.75) a^***^ | 47.20 (40.10, 53.30) a^***^ |
| **IL-13 (pg/mL)** | 26.25 (17.80, 37.94) | 28.95 (20.45, 37.10) a ^‡^ | 29.80 (22.85, 38.89) a^**^ |
| **Eotaxin (pg/mL)** | 184 (138.44, 257.85) | 215.20 (172.44, 267) a^**^ | 242 (183.81, 310.40) a^***^ |
| **IgE (IU/mL)** | 501 (177, 1139.50) | 832 (328, 1778) a^***^ | 855.75 (296, 1550.88) a^**^ |

Results are presented as median (25th percentile, and 75th percentile). The *p*-values were from the Kruskal-Wallis test followed by Dunn-Bonferroni post hoc test between each skin lesions group. ^a^ Significant difference from without skin lesions group. ^***^*p* < 0.001; ^**^*p* < 0.01; ^‡^*p* = 0.075.
