# Supplementary material for: Association between serum periostin levels and the severity of arsenic-induced skin lesions
Source: PLoS One. 2023 Jan 4;18(1):e0279893. doi: 10.1371/journal.pone.0279893 (PMC9812306; doi:10.1371/journal.pone.0279893)
Supplement: Attachment — Submitted filename: Response to Reviewers.docx [file pone.0279893.s005.docx]

We would like to thank the editor for giving us opportunity to revise the manuscript. We highly appreciate the reviewers for their constructive suggestions. Please find below our responses.

**Journal Requirements:**

1. Please ensure that your manuscript meets PLOS ONE's style requirements, including those for file naming. The PLOS ONE style templates can be found at https://journals.plos.org/plosone/s/file?id=wjVg/PLOSOne_formatting_sample_main_body.pdf and 
https://journals.plos.org/plosone/s/file?id=ba62/PLOSOne_formatting_sample_title_authors_affiliations.pdf

**Response:** Style and file naming were corrected according to the journal standard.

2. We note that Figure 1 in your submission contain copyrighted images. All PLOS content is published under the Creative Commons Attribution License (CC BY 4.0), which means that the manuscript, images, and Supporting Information files will be freely available online, and any third party is permitted to access, download, copy, distribute, and use these materials in any way, even commercially, with proper attribution. For more information, see our copyright guidelines: http://journals.plos.org/plosone/s/licenses-and-copyright.

We require you to either (1) present written permission from the copyright holder to publish these figures specifically under the CC BY 4.0 license, or (2) remove the figures from your submission:

**Response:** The Fig 1 does not contain any copyrighted images. The images presented in Fig 1, were taken by us from the individuals participated in our research. These are the images of skin lesions caused by arsenic exposure. The images show the skin lesions on the back, chest, palms and soles of the arsenic-exposed participants without any face or any kinds of individual identity. Moreover, all participants provided their written consents prior to their final enrolment as we mentioned in the “Materials and methods” section of the manuscript. Therefore, we don’t need to take any permissions from any individuals/organizations for the images used in this study.

3. Please review your reference list to ensure that it is complete and correct. If you have cited papers that have been retracted, please include the rationale for doing so in the manuscript text, or remove these references and replace them with relevant current references. Any changes to the reference list should be mentioned in the rebuttal letter that accompanies your revised manuscript. If you need to cite a retracted article, indicate the article’s retracted status in the References list and also include a citation and full reference for the retraction notice.

**Response:** We have revised the reference section. We have included some additional references which were required to revise the manuscript according to the reviewers’ comments.

**Responses to the reviewers’ comments**

**Reviewer # 1**

1. The authors should include information on the response rate for the sample collection. This information is missing from this article, as well as the earlier article that they cite in relation to sample collection.

**Response:** For this study and our two previous studies [Tony et al., 2022; Rahman et al., 2021], we could not calculate the response rate as we did not recruit any new individuals. Study participants recruitment was done in our previous study [Siddique et al., 2020] which showed the association between arsenic exposure and characteristic features of asthma. The overall response rate of that study [Siddique et al., 2020] was 92% (93% from arsenic-endemic areas, and 91% from non-endemic area). We have included this information in the “Materials and methods” section (please see lines: 127-130) of the revised manuscript.

2. What measures were adopted to quantify and eliminate recall bias of the study participants during the generation of questionnaire based demographic data?

**Response:** The demographic information collected through questionnaires were age, sex, occupation, education, personal income, and smoking and used them as variables in our study. These variables are generally known not to be affected by recall bias.

3. This manuscript will benefit from better visualization of the data rather than tabulation. As such, I suggest that the authors move tables 2 and 6 to the supplementary data and replace them with box and whisker plots showing individual data points for all samples in the main manuscript for better clarity. Additionally, they should also update the box plot in Figure 2 to show all the individual data points in each group in addition to the box plots.

**Response:** As per reviewer’s suggestion, we have included Tables 2 and 6 in the supplement and included their graphical representation in the main manuscript (please see Fig 2 and Fig 4 in the revised manuscript). Additionally, individual data points have been included in Fig 3 (Fig 2 in the previous manuscript).

**Reviewer # 2**

**Major comments**

1. The odds ratios reported for periostin seem very large. Is there prior research with regard to the association between periostin and other health outcomes that could demonstrate the plausibility of these values? Can some information be added to assist with interpretation, given that the periostin values were log-transformed?

**Response:** We used log transformed periostin value in our regression model to examine the association between periostin levels and the risk of skin lesion (Table 3 and Table 5 in the previous manuscript) to improve the estimation quality. If we don’t use log transformed values of periostin, the odds ratios will be small but corresponded well with the other studies showing the association between periostin and other health outcomes [Elhady et al., 2017; Yavuz et al., 2021; Ji et al., 2017]. We have included one statement in the discussion section regarding the similarities of our results with other studies on different health outcomes (please see lines: 334-335). Since periostin is a covariate here, normality assumption is not needed. We have revised Table 3 and Table 5 (Table 2 and Table 4 in the revised manuscript) with original (without log transformed) values of periostin.

2. Given that the mediation analysis is one of the highlighted findings in this manuscript, the methods used for the mediation analysis need to be described in more detail. There are several different methods used to perform mediation analysis, including more traditional methods (which are not recommended) as well as more advanced, sophisticated methods. At least a discussion of the possibility of residual confounding between the exposure and mediator and the mediator and outcome should be mentioned. A formal mediation analysis also requires a prospective study ideally, and this limitation should also be mentioned in the context of the mediation analysis.

**Response:** Thank you for the valuable suggestion. We represented the causal relationship using direct, and indirect (mediated through periostin) effects which were estimated using the standard Baron-Kenny regression-based approach [Valeri and Vanderweele, 2013; VanderWeele and Vansteelandt, 2014]. We have postulated a directed acyclic graph (DAG) which has been included in the revised Manuscript (Fig S2). We have provided a short description on the mediation analysis in the “Statistical analysis” section (please see lines: 197-210) of the revised manuscript. We have also included the limitation of the analysis in the discussion section (please see lines: 396-400).

**Minor comments**

1. Why not add periostin to Table 2?

**Response:** The reviewer #1 has suggested that we move Table 2 into supplementary (Table S1 in the revised manuscript) and replace them with box and whisker plots. If we add periostin data to Table S1 (Table 2 in the previous manuscript) and present them with box and whisker plots, it will be redundant to another Figure (Fig 3A in the revised manuscript). To avoid the redundancy, the periostin data have not been presented in Table S1 and Fig 2.

2. It is not described, how the covariates were modeled/controlled for. For example, was education treated in categories or continuously? It is common place to add this information to table footnotes.

**Response:** We described how the covariates were modeled in the statistical section (please see lines: 189-192 in the revised manuscript). We used education as categorical variable which was mentioned in the statistical section (please see lines: 177-179 in the revised manuscript). We also added this information to the table footnotes (Table 2, 3, and 4 in the revised manuscript).

3. Please use a consistent number of decimal places in text and tables. Even if the final decimal place is a zero, it should be included to show the degree of precision.

**Response:** We have revised the text and tables according to the suggestion of the reviewer.

**Typos**

1. Table four, odds ratio should be “odds ratios”

**Response:** We have fixed the error (Table 3 in the revised manuscript).

2. Bottom of p. 10 (line 218), n=112 represents ¼ of the total participants, not ¼ of the participants with skin lesions.

**Response:** We have revised it (please see lines: 230-231 in the revised manuscript).

3. A 2.3-fold increase (line 248) does not correspond to an odds ratio of 9.87.

**Response:** We have revised it (please see line: 259 in the revised manuscript).

4. Line 265, “lesions in low-exposure area” should be “lesions in the low-exposure area.

**Response:** We have revised it (please see line: 277 in the revised manuscript).

5. Line 281, “…without skin lesions, early-stage and advanced-stage…” should be “…without skin lesions, to those with early-stage and advanced-stage…”

**Response:** We have revised it (please see line: 292 in the revised manuscript).

6. No comma is needed after “approximately” in line 389.

**Response:** We have removed the comma (Please see line: 405 in the revised manuscript).
